# Neuromodulation and neural networks in psychiatric disorders: current status and emerging prospects

**DOI:** 10.1017/S003329172510158X

**Published:** 2025-09-26

**Authors:** Panayiota G. Michalopoulou, Kyrillos M. Meshreky, Zoe Hommerich, Sukhi S. Shergill

**Affiliations:** 1 Department of Psychosis Studies, Institute of Psychiatry, Psychology and Neuroscience (IoPPN), King’s College London, London, UK; 2 South London and Maudsley NHS Foundation Trust, London, UK; 3 School of Psychology, Cardiff University, Cardiff, UK; 4 Department of Clinical Psychological Science, Maastricht University, Maastricht, The Netherlands; 5 Kent and Medway Medical School, Canterbury, UK; 6 Kent and Medway NHS and Social Care Partnership Trust, Maidstone, UK

**Keywords:** Neural networks, Non-invasive brain stimulation (NIBS), Transcranial magnetic stimulation (TMS), Transcranial direct current stimulation (tDCS), Transcranial alternating current stimulation (tACS), Focused ultrasound stimulation (FUS), Psychiatric disorders, Depression, Schizophrenia, Obsessive-compulsive disorder, Cognition, Biomarkers, Precision psychiatry

## Abstract

Psychiatric disorders lead to disability, premature mortality and economic burden, highlighting the urgent need for more effective treatments. The understanding of psychiatric disorders as conditions of large-scale brain networks has created new opportunities for developing targeted, personalised, and mechanism-based therapeutic interventions. Non-invasive brain stimulation (NIBS) techniques, such as transcranial magnetic stimulation (TMS) and transcranial direct current stimulation (tDCS), can directly modulate dysfunctional neural networks, enabling treatments tailored to the individual’s unique functional network patterns.

As NIBS techniques depend on our understanding of the neural networks involved in psychiatric disorders, this review offers a neural network-informed perspective on their applications. We focus on key disorders, including depression, schizophrenia, and obsessive-compulsive disorder, and examine the role of NIBS on cognitive impairment, a transdiagnostic feature that does not respond to conventional treatments. We discuss the advancements in identifying NIBS response biomarkers with the use of electrophysiology and neuroimaging, which can inform the development of optimised, mechanism-based, personalised NIBS treatment protocols.

We address key challenges, including the need for more precise, individualised targeting of dysfunctional networks through integration of neurophysiological, neuroimaging and genetic data and the use of emerging techniques, such as low- intensity focused ultrasound, which has the potential to improve spatial precision and target access. We finally explore future directions to improve treatment protocols and promote widespread clinical use of NIBS as a safe, effective and patient-centred treatment for psychiatric disorders.

## Introduction

Psychiatric disorders are a leading cause of global disability, accounting for 32.4% of years lived with disability and 13% of disability-adjusted life years, comparable to cardiovascular and circulatory diseases (Vigo, Thornicroft, & Atun, 2016). Medications and psychotherapies offer modest symptom improvements but do not significantly alleviate disability or improve functional outcomes (Leichsenring, Steinert, Rabung, & Ioannidis, 2022). Additionally, 20%–60% of individuals fail to respond adequately to optimal treatments (Howes, Thase, & Pillinger, 2022), and medications often have side effects that limit adherence and acceptability. The economic burden is substantial, with mental disorders costing £300 billion in England in 2022 through premature mortality, direct losses to the economy through unemployment, and indirect losses related to health and care costs (Cardoso & McHayle, 2024). These highlight the urgent need for novel, mechanism-based, safe, effective, and acceptable treatments as alternatives or additions to existing treatments to enhance functional outcomes and quality of life of people with mental disorders.

Psychiatric disorders are increasingly understood as conditions of large-scale brain networks rather than abnormalities within isolated brain regions. These large-scale brain networks are neural systems distributed across most of the brain, anatomically interconnected and functionally synchronized, and support the necessary cognitive, emotional, and sensorimotor processes underpinning complex human behaviors. Dysfunctional information processing within and between these networks is thought to contribute to the pathophysiology of psychiatric disorders and the manifestation of their symptoms (Menon, 2011; Sporns, 2014).

**Figure 1:**
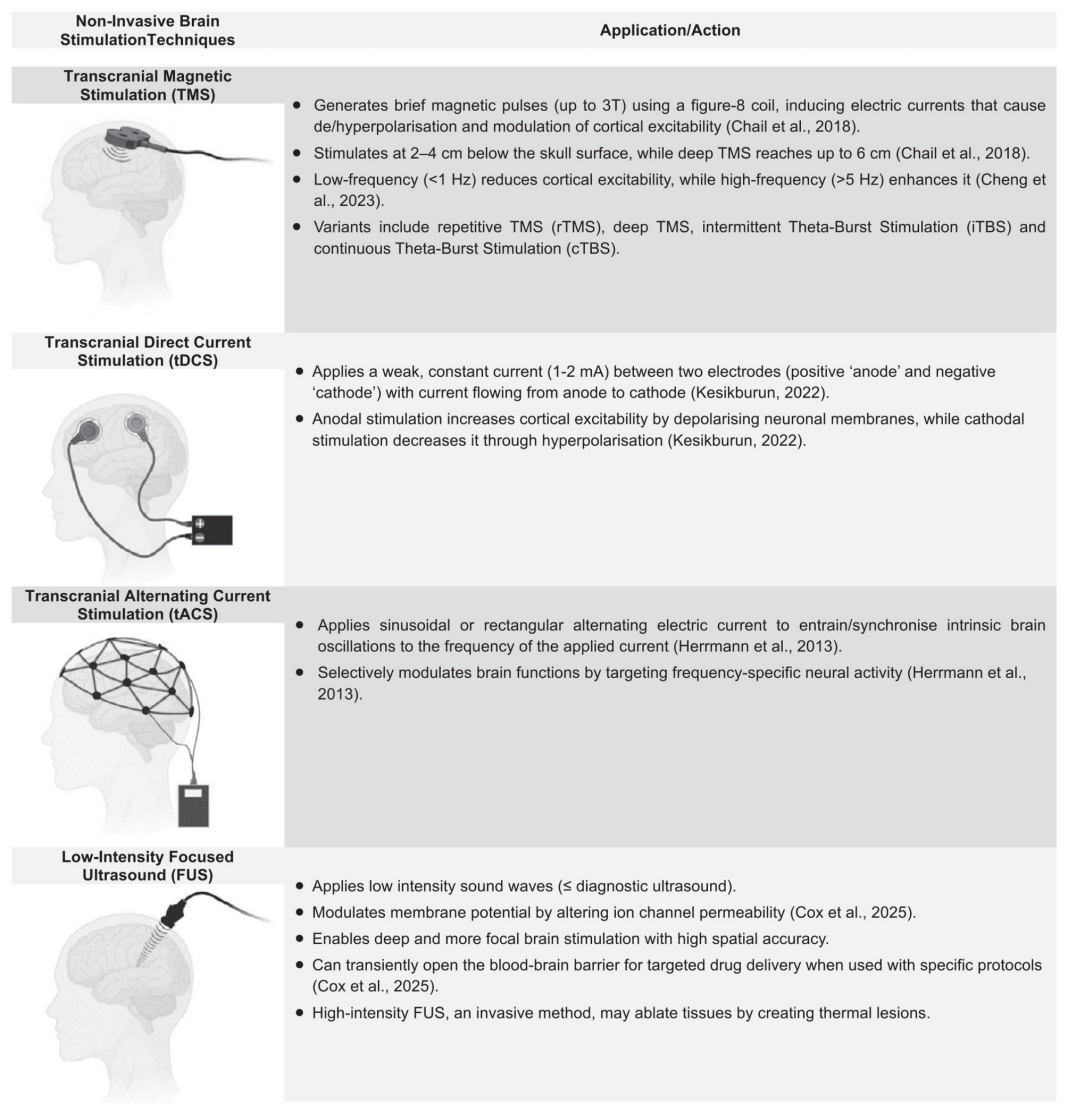
Non-invasive brain stimulation techniques and their mode of application and action.

In this context, modulation of specific brain networks through externally applied electro-magnetic stimulation (collectively known as ‘neuromodulation’ or ‘neurostimulation’) is used to directly modify ‘abnormal’ neural network activity in psychiatric disorders. While medications and psychotherapies indirectly modulate neural activity (Celada, Puig, & Artigas, 2013; Schrammen et al., 2022), ‘neuromodulation’ offers the opportunity for a more selective, targeted network-based approach, which has not been feasible so far with medications and psychotherapies. Furthermore, unlike traditional treatments, ‘neuromodulation’ enables personalized interventions based on an individual’s unique brain network dysfunction. ‘Neuromodulation’ includes invasive and non-invasive brain stimulation (NIBS) techniques, with the latter extensively used for research and treatment in psychiatric disorders, particularly in treatment-resistant disorders (e.g. depression) and difficult-to-treat specific symptoms (e.g. negative symptoms and cognitive impairment) (Figure 1).

Both invasive and NIBS techniques depend on our understanding of brain networks involved in psychiatric disorders. Functional neuroimaging techniques, particularly resting state functional MRI (rsfMRI) functional connectivity (FC) analyses, are the most widely used techniques for the identification of large-scale network abnormalities in psychiatric disorders. FC measures temporal correlations in activity between spatially distant brain regions, revealing spontaneous activity patterns without task-related interference, and has led to the identification of core brain networks, which are hypothesized to play key roles in psychiatric disorders (Yeo et al., 2011) (Table 1).

Findings from large-scale neuroimaging databases have challenged the classical views that specific disorders map onto distinct brain regions or even networks and suggest overlap of neural networks in psychiatric disorders, along with more disorder-specific effects. For example, the ‘triple network model for psycho-pathology’ proposes that DMN, Frontoparietal Network (FPN)/Central Executive Network (CEN), and Salience Network (SN) are implicated in multiple psychiatric disorders (Menon, 2011). SN (Downar, Blumberger, & Daskalakis, 2016; Segal et al., 2023) and the Limbic Network (LIN) (Ishida et al., 2023) have also been proposed as ‘common core’ neural networks for psychiatric disorders. The involvement of common neural networks across psychiatric disorders has been suggested as ‘transdiagnostic’ biomarkers, while the involvement of additional neural networks in each disorder may contribute to the phenotypic differences among psychiatric disorders (Chavez-Baldini et al., 2023; Segal et al., 2023). This is particularly significant for NIBS, as it implies that single brain targets for each disorder may be insufficient and points to the integration of psychiatric diagnosis, individual symptom profiles, and brain networks to improve our understanding of the pathophysiology of disorders and develop tailored NIBS interventions.

In this review, we offer a neural network-informed perspective on NIBS applications. We focus on key disorders, including depression, schizophrenia, and obsessive-compulsive disorder, and examine the impact of NIBS on cognitive impairment, a transdiagnostic feature that does not respond to conventional treatments. We discuss the advancements in identifying NIBS response biomarkers with the use of electrophysiology and neuroimaging, and conclude with key challenges and future directions to improve treatment protocols and promote widespread clinical use of NIBS as a safe, effective, and patient-centred treatment for psychiatric disorders.

### Major depressive disorder

Major depressive disorder (MDD) is a highly heterogeneous syndrome affecting 320 million people globally and is a leading cause of disability (World Health Organization, 2017). Around 30% of patients develop treatment-resistant depression (TRD), defined as non-response to two adequate antidepressant trials (McIntyre et al., 2023). TRD was the first psychiatric disorder for which a NIBS treatment was approved, with rTMS over the left dorsolateral prefrontal cortex (lDLPFC) receiving FDA clearance in 2008. The UK’s NICE guidelines also recommend rTMS for TRD (National Institute for Health and Care Excellence, 2015).

Depression is currently conceptualized as a systems-level disorder caused by disrupted network regulation under cognitive, emotional, or physical stress (Mayberg, 2003). The key brain networks associated with MDD include:

- Affective Network (AN) with increased FC between AN regions, which is associated with excessive negative feelings (Li et al., 2018), while the onset of depression is associated with increased FC between amygdala and subgenual ACC (sgACC) (Davey et al., 2015) and the strength of connectivity between sgACC and dorsomedial frontal cortex correlates with the severity of depression (Davey, Harrison, Yücel, & Allen, 2012). Hypoconnectivity between AN and the prefrontal cortex (PFC) may underlie top-down emotion regulation deficits in MDD (Kaiser, Andrews-Hanna, Wager, & Pizzagalli, 2015).
- Salience Network (SN) with recent neuroimaging evidence showing almost twice as large SN in MDD compared to controls, including the regions of anterior insula, ACC, and lateral PFC. This enlargement precedes clinical depression in children who later develop depression, suggesting a potential risk biomarker (Lynch et al., 2024). During a depressive episode, changes in FC between SN nodes, particularly nucleus accumbens (NAc) with ACC and anterior insula, predict the emergence and remission of anhedonia and anxiety, respectively (Lynch et al., 2024).
- Reward Network (RN) with reduced connectivity between the regions of RN, which is associated with diminished motivation, interest, and anhedonia (Li et al., 2018). MDD is also associated with activation imbalances between RN (ventral striatum – reduced activation) and AN (Orbitofrontal Cortex – OFC – increased activation) during reward processing tasks (Ng, Alloy, & Smith, 2019).
- Default Mode Network (DMN) with hyperconnectivity within DMN nodes, which has been suggested to underlie excessive rumination in MDD, as an impairment of DMN-related self-referential mental activity (Kaiser et al., 2015).
- Central Executive Network (CEN) with increased DMN-CEN connectivity, which is viewed as DMN suppressing CEN and leading to a bias towards internal self-referential/ruminative thoughts (Young et al., 2023). Additionally, increased connectivity between CEN and the dorsal attention network has been found and is thought to be associated with diminished attention towards the external environment in MDD (Kaiser et al., 2015). CEN also shows increased connectivity with subcortical structures, such as the hippocampus, which may be related to the biased focus on unpleasant memories in MDD (Young et al., 2023). fMRI studies have shown DLPFC underactivation and VMPFC overactivation in depression (Koenigs & Grafman, 2009), while recovery is linked with ‘normalization’ of this pattern (Brody et al., 2001). The ‘prefrontal asymmetry’ theory is one of the classic theories in MDD, based on fMRI evidence of hypoactivity of the left and hyperactivity of the right DLPFC (rDLPFC) (Bruder, Stewart, & McGrath, 2017).

Brain lesion, TMS, and DBS studies identified a shared neural network for depression, including DLPFC, sgACC, and ventromedial prefrontal cortices (vmPFC). These regions align with the CEN and dorsal attention network (DAN) and correlate negatively with DMN and limbic networks (Siddiqi et al., 2021). These findings suggest that depression symptoms, whether caused by a primary psychiatric disorder (e.g. MDD) or structural brain lesions, may share common brain networks and highlight the potential of NIBS in studying and treating transdiagnostic psychiatric symptoms.

### NIBS for MDD treatment

rTMS is the NIBS technique with the most robust evidence for clinical efficacy and treatment effect estimates for MDD, while evidence for the use of tDCS is evolving.

lDLPFC is the primary target for rTMS and tDCS using ‘excitation’ protocols to target ‘prefrontal asymmetry’. It is assumed that high-frequency (HF >5 Hz) rTMS (HF-rTMS) induces cortical excitation, while low-frequency (LF 1 Hz) rTMS (LF-rTMS) induces inhibition (Pascual-Leone, Valls-Solé, Wassermann, & Hallett, 1994). Standard rTMS uses 10 Hz on lDLPFC, while tDCS applies 1–2 mA currents, both of which increase local cortical excitability (Brunoni et al., 2016).

Meta-analyses confirm the efficacy of rTMS as monotherapy and adjunctive treatment for depression (Brunoni et al., 2017; Vida et al., 2023). Around 40% of TRD patients respond to rTMS versus 10% to sham, with remission rates of 36% versus 8% (Vida et al., 2023). rTMS is well-tolerated, acceptable, and cost-effective compared to multiple medication trials (Nguyen & Gordon, 2015; Voigt, Carpenter, & Leuchter, 2017). However, rTMS response rates vary widely, prompting protocol modifications such as bilateral DLPFC stimulation (HF on lDLPFC, and LF on rDLPFC) and priming (HF-rTMS before LF-rTMS) to optimize effects (Fitzgerald et al., 2008). Network analyses favor priming, bilateral rTMS, and bilateral theta burst stimulation, while accelerated, synchronized, and deep rTMS show no advantage over sham (Brunoni et al., 2017; Mutz et al., 2019; Shi et al., 2024).

tDCS in depression shows response and remission rates of 34% and 23%, respectively (Brunoni et al., 2016). Efficacy declines with treatment resistance (Brunoni et al., 2016; Mutz et al., 2019) and improves with longer sessions (Brunoni et al., 2016). A recent multisite home-based RCT found 2–3 times higher response and remission rates versus sham (Woodham et al., 2025). High acceptability, safety, portability, and cost-effectiveness position tDCS as a potential first-line depression treatment (Woodham et al., 2025).

Since depression symptoms have been shown to share neural networks regardless of their cause (Siddiqi et al., 2021), this suggests that both unipolar and bipolar depression should respond to similar NIBS protocols. Meta-analyses show that this may indeed be the case, with rTMS showing small but significant improvements in bipolar depression (Tee & Au, 2020). However, polarity-specific analyses found rTMS effective for unipolar but not bipolar depression (Hyde et al., 2022). Additionally, an iTBS trial targeting the left DLPFC in bipolar depression showed no efficacy (McGirr et al., 2021). In contrast, a more recent iTBS trial in bipolar depression using personalized lDLPFC targeting based on the FC between the sgACC and lDLPFC reported significant clinical improvements (Appelbaum et al., 2025). These conflicting findings emphasize the importance of personalized targeting to optimize the clinical efficacy of rTMS and to clarify whether rTMS can effectively treat bipolar depression or whether distinct pathophysiological patterns differentiate two similar phenotypes (unipolar and bipolar depression), requiring disorder-specific NIBS treatment protocols.

### NIBS biomarkers

Combining TMS/tDCS with EEG and neuroimaging has shown potential for the identification of biomarkers of treatment response, enabling patient stratification and individualized treatment protocols to optimize treatment response.

### EEG biomarkers

EEG predicts rTMS response more accurately than antidepressant response, as it better captures neural activity in targeted cortical networks (Watts et al., 2022), while being accessible, tolerable, and cost-effective. Individual Alpha Peak Frequency (IAPF) is the frequency of the strongest alpha oscillation (7–13 Hz). Patients with IAPF near 10 Hz show higher remission rates with 10 Hz lDLPFC rTMS, while those with higher IAPF respond better to 1 Hz right DLPFC rTMS (Voetterl et al., 2023), highlighting the potential of IAPF to stratify patients to more effective rTMS protocols based on individual pre-treatment oscillatory activity. Task-Induced Frontal-Midline Theta Power reflects task-induced rostral ACC (rACC) activity, a key hub of DMN, which plays an important role in depression pathophysiology. Changes in frontal-midline theta power following rTMS may differentiate responders from non-responders (Bailey et al., 2018; Li, et al., 2016).

### Neuroimaging and TMS-EEG biomarkers

Dysfunctional sgACC is central to the pathophysiology of depression. It shows increased activity with reciprocal decreased rDLPFC activity during depressive episodes, with reversal of this pattern during depression recovery (Mayberg et al., 1999). Evidence suggests that DLPFC-sgACC connectivity may be a marker of rTMS in depression. TMS stimulation of the lDLPFC regions, which were more negatively correlated (‘anti-correlated’) with sgACC showed better clinical efficacy in MDD (Fox, Buckner, White, Greicius, & Pascual-Leone, 2012), highlighting the potential for the development of FC-based biomarkers to optimize clinical outcomes. More recently, computational models have been developed in large-scale FC datasets to enable FC-guided (sgACC-lDLPFC) personalization of rTMS in depression (Cash et al., 2021) and have recently been used in an iTBS trial in bipolar depression with positive results, as discussed above (Appelbaum et al., 2025). Combining TMS with electroencephalography (TMS-EEG) showed increased sgACC excitability and stronger effective connectivity between the sgACC and lDLPFC in depression, both of which decreased after rTMS treatment over the lDLPFC, and the reduction in connectivity correlated with symptom improvement (Hadas et al., 2019). Lower baseline glutamate in ACC is associated with better rTMS response (Gonsalves et al., 2024). tDCS was more effective in MDD patients with higher pre-treatment activation levels of the left PFC (Nord et al., 2019) and larger left PFC volumes (Bulubas et al., 2019).

### Schizophrenia

Schizophrenia (SCZ) is a severe mental disorder affecting 1% of the population and characterized by significant heterogeneity in symptom presentation, treatment response, and prognosis. Current evidence suggests a multifactorial etiology involving neurodevelopmental, genetic, and environmental factors (Murray, Bhavsar, Tripoli, & Howes, 2017).

SCZ symptoms are grouped into positive, negative, and cognitive clusters, and empirical evidence from rsfMRI studies supports the ‘**disconnection hypothesis**,’ which links symptoms to altered FC between PFC, subcortical (e.g. thalamic), and associative cortical (e.g. temporal) regions (Friston, Brown, Siemerkus, & Stephan, 2016; Friston & Frith, 1995). Hypoconnectivity is particularly evident in the frontal brain (Pettersson-Yeo, Allen, Benetti, McGuire, & Mechelli, 2011). Concurrent hypo- and hyper-connectivity patterns have been shown with reduced connectivity between DLPFC-limbic cortices and the mediodorsal thalamus and increased connectivity between primary-sensorimotor cortices and ventral thalamic nuclei. These FC alterations have been associated with SCZ symptoms (e.g. Anticevic et al., 2015).

Positive symptoms in SCZ correlate with hyperconnectivity of the primary-sensorimotor cortices to thalamic and striatal nuclei (Avram, Brandl, Bäuml, & Sorg, 2018). AVHs correlate with hyper-connectivity in the left auditory cortex and increased activity within the left temporoparietal cortex alongside reduced prefrontal top-down control (Shao, Liao, Gu, Chen, & Tang, 2021; Shergill, Brammer, Williams, Murray, & McGuire, 2000). Negative symptoms have long been linked to dysfunctional PFC (Liddle, 1987), with functional neuroimaging studies showing associations with DLPFC and ventrolateral prefrontal cortex (VLPFC) activity (Goghari, Sponheim, & MacDonald, 2010). Negative symptoms have also been associated with altered FC between DLPFC and DMN-cerebellar circuits (Brady et al., 2019). Patients with SCZ and prominent avolition show disrupted FC between the ventral tegmental area (VTA) (a key source of mesocorticolimbic dopamine involved in reward and motivation) and cortical regions related to value processing and action selection, such as the bilateral VLPFC, insular cortex, lateral occipital cortex, and DLPFC (Giordano et al., 2018).

While the underlying causes of brain functional dysconnectivity in SCZ remain unclear, an optimal balance between excitatory (glutamate-mediated) and inhibitory (GABA-mediated) systems is critical for regulating information processing within and between neural networks (Turrigiano & Nelson, 2004). Disruption of the Excitation/Inhibition (E/I) balance is linked to SCZ pathophysiology, the lack of response of negative and cognitive symptoms to antipsychotics, as well as treatment resistance, which is observed in approximately 30% of patients (Howes & Shatalina, 2022).

TMS is uniquely placed for studying E/I balance and connectivity. Combined with electromyography (EMG), it enables non-invasive assessment of E/I indices via standardized primary motor cortex (M1) protocols, serving as a proxy for cortical dysfunction. A meta-analysis of TMS-EMG studies in SCZ found significant inhibition deficits, as measured by Short Interval Cortical Inhibition (SICI) (*d* = 0.62), supporting the E/I imbalance hypothesis and showing potential as a diagnostic and treatment biomarker (Lányi et al., 2024). TMS-EEG, which extends the methodology beyond M1, shows potential as a treatment response biomarker. This has been demonstrated in epilepsy (Gefferie et al., 2023) and is currently being investigated in SCZ (Di Hou, Santoro, Biondi, Shergill, & Premoli, 2021; Santoro et al., 2024).

### NIBS treatments for Schizophrenia

NIBS has been used to treat treatment-resistant symptoms, including persistent positive (primarily AVHs) but also negative and cognitive symptoms, which do not respond to current treatments (Fusar-Poli et al., 2015).

### AVHs

Most NIBS trials for AVHs apply left temporoparietal area (TPA) inhibition and frontal activation protocols based on evidence that therapeutic effects may result from the normalization of hyperconnectivity and increased activity in the left auditory cortex/TPA, as well as the restoration of the diminished top-down control from the PFC (Gromann et al., 2012). Typically, rTMS studies apply low-frequency rTMS (inhibition) (1 Hz) (LF-rTMS) to lTPA, while tDCS studies apply concurrent cathodal stimulation (inhibition) to lTPA and anodal stimulation (activation) to lDLPFC.

The treatment effects of both techniques are significant but small, ranging between 0.19 and 0.49 for rTMS (He et al., 2017; Hyde et al., 2022; Li, Cao, Liu, Li, & Xu, 2020; Otani, Shiozawa, Cordeiro, & Uchida, 2015; Slotema, Blom, Van Lutterveld, Hoek, & Sommer, 2014), with some trials reporting negative results (Li et al., 2020), and 0.42 for tDCS (Hyde et al., 2022). Both techniques have good tolerability with no significant differences in attrition rates between active and sham treatments (Slotema, Aleman, Daskalakis, & Sommer, 2012; Valiengo et al., 2020). The efficacy of rTMS on other positive symptoms, particularly delusions, is less robust and more variable across studies (Kennedy, Lee, & Frangou, 2018).

Combining neuroimaging with NIBS has highlighted the role of the left temporoparietal network in treatment response. Higher blood flow in the left superior temporal gyrus (STG) predicts rTMS response for AVHs (Homan, Kindler, Hauf, Hubl, & Dierks, 2012), while left STG FC predicts tDCS response for AVHs (Paul et al., 2022). Pre-treatment FC alterations in STG and decreased Degree Centrality (DC), which quantifies the magnitude of neural activity in a specific brain region relative to overall brain activity (Tomasi, Shokri-Kojori, & Volkow, 2016), in prefrontal and occipital cortices reverse post-treatment and correlate with symptom improvement (Xie et al., 2023).

### Negative symptoms

Overall, NIBS has shown promising effects on negative symptoms, which are typically resistant to standard treatments and have a substantial impact on the functional outcomes and prognosis of SCZ (Rabinowitz et al., 2012).

Meta-analyses of rTMS RCTs showed significant small (0.41) to medium (0.64) effect sizes (Aleman, Enriquez-Geppert, Knegtering, & Dlabac-de Lange, 2018; Lorentzen, Nguyen, McGirr, Hieronymus, & Østergaard, 2022) compared to sham and significant small effects for tDCS (0.50) (Aleman et al., 2018). The most common targeted area across studies is the lDLPFC, with HF being the most efficacious for both rTMS (Lorentzen et al., 2022) and tDCS (Yu et al., 2020). A recent meta-analysis found that iTBS on the left dorsal PFC was effective for negative symptoms (Tan et al., 2023).

FC patterns in early SCZ have shown that greater negative symptom severity correlates with reduced rDLPFC connectivity to a network spanning cerebral and cerebellar DMN nodes, with the midline cerebellar node being the strongest predictor of symptom severity. rTMS targeting this region led to both symptomatic improvement and enhanced DLPFC-cerebellar FC, indicating a mechanism of clinical benefits (Brady et al., 2019). FC between VTA and DLPFC could be explored for personalized DLPFC targeting and prediction of treatment response in patients with prominent avolition (Giordano et al., 2018). Beyond FC patterns, structural markers such as pre-treatment grey matter density reductions in the prefrontal, insular, medial temporal, and cerebellar cortices, alongside increases in parietal and thalamic structures, have also been linked to rTMS response in predominantly negative SCZ (Koutsouleris et al., 2018).

### Obsessive-compulsive disorder

Obsessive-compulsive disorder (OCD) is a chronic, heterogeneous disorder affecting 1%–4% of the population. Standard treatments include SSRIs and psychotherapies, but 30% of cases are treatment-resistant, affecting functional outcomes and quality of life (National Institute for Health and Care Excellence, 2024) and emphasizing the need for more effective treatments.

Traditionally, OCD has been associated with dysfunctional cortico-striato-thalamo-cortical (CSTC) networks (Alexander & Crutcher, 1990), resulting in hyperactive OFC-ventromedial caudate networks and hypoactive executive networks, including DLPFC and dorsolateral caudate.

FC studies showed altered connectivity within CSTC, including (a) dysconnectivity between striatal and cortical networks (i.e. caudate hyperconnectivity with the fronto-limbic network and hypoconnectivity with frontoparietal network regions, along with NAc hypoconnectivity with fronto-limbic network regions); (b) hyperconnectivity between thalamus and striatum (putamen and caudate); and (c) dysconnectivity between ACC and frontolimbic networks (Liu et al., 2022). The dorsal ACC, which is considered a ‘hub’ of OCD with dense connections to ventral affective and dorsal cognitive networks, is involved in cognitive control (CC) impairments in OCD and shows hyperactivity in rsfMRI studies (McGovern & Sheth, 2017).

Current OCD models have proposed the following networks in OCD (Shephard et al., 2021; van den Heuvel et al., 2016):

- The fronto-limbic network, which includes the amygdala and vmPFC, is involved in emotional responses, such as fear and anxiety, and shows aberrant activation in OCD during emotional processing tasks (Thorsen et al., 2018).
- The Sensorimotor Network (SMN). In the largest FC whole-brain analysis in OCD to date (Bruin et al., 2023), SMN showed the most significant FC hypoconnections among OCD brain networks. SMN has been linked with ordering, arranging, counting, and repeating compulsions and with sensory perceptions of ‘feeling dirty’ and associated washing and cleaning compulsions (Shephard et al., 2021). Among SMN nodes, fMRI studies have shown middle cingulate cortex hypoactivation and bilateral ACC hyperactivation in OCD with prominent washing (Yu et al., 2022) and supplementary motor area (SMA) hyperactivity, possibly reflecting response inhibition impairment in OCD (de Wit et al., 2012).
- The ventral cognitive network, which involves the inferior frontal gyrus (IFG), VLPFC, ventral caudate, and thalamus, and mediates response inhibition. IFG and caudate activity are reduced during inhibition tasks in OCD (Abramovitch, Abramowitz, & Mittelman, 2013).
- The ventral affective network, involving OFC, NAc, and thalamus, regulates reward processing. fMRI studies show hypoactivation of the NAc and OFC during reward anticipation and decision-making in OCD (Figee et al., 2011; Norman et al., 2018), along with hypoconnectivity between these regions, which correlates with more severe symptoms (Liu et al., 2022), suggesting reward-related impairments in OCD.
- The dorsal cognitive network, which is connected to CEN and is involved in top-down control of emotional, motor, and cognitive processes, such as response inhibition and cognitive flexibility, and working memory. It includes DLPFC, dorsomedial prefrontal cortex (dmPFC), dorsal caudate, thalamus, and pre-SMA and is suggested to contribute to OCD through executive dysfunction (Shephard et al., 2021).
- The triple model networks (DMN, FPN, SN) exhibit hypoconnectivity in OCD, potentially related to difficulties switching between repetitive thoughts and goal-directed actions (Bruin et al., 2023). Alteration in error-processing is a robust finding in OCD and is associated with increased activity in SN, DMN, SMN, and fronto-limbic networks and has been proposed as an endophenotype for OCD (Riesel et al., 2019).

### NIBS treatments for OCD

Deep rTMS, which penetrates deeper brain structures compared to traditional TMS, received FDA approval for OCD in 2018 with HF (20 Hz) bilateral medial PFC/ACC stimulation, showing a 38% response rate versus 11% for sham. This was followed by approval of HF bilateral deep rTMS over dmPFC in 2020. NICE considers the evidence insufficient to recommend rTMS for OCD (National Institute for Health and Care Excellence, 2020).

rTMS is effective for OCD, with effect sizes ranging from small (0.43) to large (0.79) with high heterogeneity in most studies (Kar, Agrawal, Silva-dos-Santos, Gupta, & Deng, 2024), with deep TMS being superior to traditional (Suhas et al., 2023). DLPFC, pre-SMA, and OFC have also been targeted in OCD studies, but evidence remains inconclusive due to small samples and protocol variabilities (Grassi, Moradei, & Cecchelli, 2023).

FDA-approved OCD protocols involved HF (‘excitatory’) rTMS, despite targeting hyperactive CSTC networks. This may seem paradoxical, as LF (‘inhibitory’) protocols would be expected to induce therapeutic effects in this case. Accumulating evidence suggests that the distinction between HF-excitatory/LF-inhibitory stimulation may be oversimplified. For example, in smokers, HF and not LF rTMS to the hyperactive insula reduced cigarette consumption (Dinur-Klein et al., 2014). HF rTMS acts as a neuro-modulator and not just as an excitatory tool, potentially ‘resetting’ dysregulated networks through synaptic plasticity changes, altered inhibitory interneuron activity, modified oscillatory patterns, and restored FC (Fitzsimmons, Oostra, Postma, Van Der Werf, & Van Den Heuvel, 2024). However, both HF and LF are effective in OCD, though iTBS, an excitatory protocol, has not shown efficacy (Kar et al., 2024). Research is needed to understand this lack of clinical benefit and further explore excitatory/inhibitory rTMS protocols. On the other hand, tDCS results are inconsistent, with some studies showing improvement (Xie et al., 2024) and others showing no effects (Pinto et al., 2022).

Treatment biomarkers for rTMS in OCD are under investigation. SMN and SN may have potential as treatment biomarkers. SMN shows the most significant hypoconnections in OCD and its error-related activity has been associated with treatment response in CBT with higher levels of pre-treatment activity predicting better response (Grützmann et al., 2022). Increases in FC between the SMN and DMN correlated with symptomatic improvement in a small tDCS clinical trial (Echevarria et al., 2024). Hypoconnectivity between SN and frontoparietal networks and increased SN activity in activation studies have also been consistently shown in OCD (Perera, Gotsis, Bailey, Fitzgibbon, & Fitzgerald, 2024).

### Cognitive impairment

Cognitive impairment (CI) is a common feature across multiple psychiatric disorders. A recent systematic review of meta-analyses of neurocognitive studies showed impaired cognition across all psychiatric disorders, indicating CI as a transdiagnostic feature. Most disorders show small to medium effect sizes of impairment across cognitive domains, while SCZ and bipolar disorder typically exhibit larger effect sizes (Abramovitch, Short, & Schweiger, 2021). CI significantly impairs functional outcomes, particularly in psychotic disorders, and conventional treatments offer little benefit, highlighting the need for more effective treatments (Sheffield, Karcher, & Barch, 2018).

In line with neurocognitive evidence, current neuroimaging evidence suggests a unifying network model for CI across psychiatric disorders. rsfMRI meta-analysis showed common FC alterations in the ‘triple network model’ associated with CI across eight psychiatric disorders (including SCZ, Bipolar Disorder, Depression, and OCD), with hypoconnectivity between DMN and ventral SN and between SN and FPN, and hyperconnectivity between DMN and FPN and between DMN and dorsal SN (Sha, Wager, Mechelli, & He, 2019). In a meta-analysis of fMRI studies in SCZ, unipolar and bipolar depression, anxiety disorders, and substance use, transdiagnostic abnormal activation was found in SN areas, including left PFC, anterior insula, right VLPFC, right intraparietal sulcus, mid-cingulate/pre-SMA, and dorsal ACC (McTeague et al., 2017). The triple model networks are involved in cognitive control (CC), the ability to regulate goal-directed behavior flexibly and adaptively in response to changing environmental demands, and CC has been suggested to underlie CI across psychiatric disorders (McTeague, Goodkind, & Etkin, 2016; Menon, 2020).

So far, the effects of NIBS on cognitive symptoms are rather inconsistent and appear to be domain-specific. For example, improvements in working memory and executive functions have been shown with corresponding changes in frontal cortical activity in a combined tDCS-fMRI study (Orlov et al., 2017). A recent meta-analysis found small but significant transdiagnostic effects of TMS and tDCS on working memory, with tDCS also improving attention/vigilance across brain disorders (including SCZ depression, dementia, Parkinson’s disease, stroke, traumatic brain injury, and multiple sclerosis), with no significant differences among disorders (Begemann, Brand, Ćurčić-Blake, Aleman, & Sommer, 2020). Combining tDCS with cognitive training showed significant longer-term improvements on working memory (Orlov et al., 2017) and stochastic learning in SCZ (Orlov et al., 2022). A recent systematic review and meta-analysis showed small yet significant improvements in attention and working memory in neurological and psychiatric disorders, including SCZ (Burton et al., 2023). lDLPFC is the most common target in rTMS (Jiang et al., 2019), while tDCS studies commonly apply anodal stimulation of lPFC/lDLPFC with various cathodal placements (Stuchlíková & Klírová, 2022).

The unifying model of CI across psychiatric disorders highlights CC as a key target for NIBS treatments and cognitive training. CC impairments have also been linked to persistent psychotic symptoms (Horne et al., 2022) and treatment-resistant SCZ (Horne et al., 2021), and combining NIBS with cognitive training targeting CC may also offer a promising approach for difficult-to-treat SCZ.

### Concluding remarks and emerging prospects

Understanding psychiatric disorders as brain network-based conditions has created new opportunities for targeted, personalized, and mechanism-based therapeutic interventions. The growing body of NIBS research in psychiatric disorders has recognized the variability in its response and is evolving to develop novel treatment protocols and identify biomarkers of response. However, there is a corpus of challenges to widespread therapeutic use.

A major challenge is precision in brain targeting, which depends on our understanding of the neural networks implicated in psychiatric disorders and on patient-specific patterns of network dysfunction. At the disorder level, as discussed above, neuroimaging studies have shown both common and distinct neural networks involved in psychiatric disorders, highlighting the importance of exploring the effects of targeting both for effective treatments. At the patient level, adapting NIBS protocols based on individual network dysfunction patterns has improved outcomes. For example, as discussed above, stimulation of lDLPFC regions, which were negatively correlated with sgACC showed better clinical efficacy in unipolar and bipolar depression (Appelbaum et al., 2025; Fox et al., 2012; Hadas et al., 2019). This suggests that a ‘one brain site fits all’ approach, using a single brain target for all patients with a specific disorder, is unlikely to further improve treatment effectiveness. A more comprehensive understanding of patient-specific brain changes, and their network context, will be necessary to develop more effective, and personally tailored, interventions. To this end, the combination of NIBS with neuroimaging/TMS methods is essential for meaningful research in the network abnormalities at the patient level and the mechanisms of treatment response. Furthermore, the inclusion of mechanistic studies in the treatment trials (e.g. EEG, MRI) is essential to explore the mechanisms of action of NIBS and their association with symptomatic improvements.

Precision in brain targeting can be improved with emerging NIBS techniques, such as low-intensity focused ultrasound (FUS) (Figure 1), which allows for deeper and more targeted stimulation of both cortical and subcortical brain regions with millimetre precision relative to TMS and tDCS. Though still in early research stages, FUS has shown promising results in preliminary trials for depression, SCZ, and anxiety (Shi & Wu, 2025).

While neuroimaging, especially rsFC, has revealed network abnormalities in psychiatric disorders and informed personalized NIBS protocols, it remains unclear whether these abnormalities are causes or consequences of the disorders since rsFC is inherently correlational. Integrating genetic and rsFC data can clarify causal links and inform treatment targets. In this context, a recent study integrating genetic and rsfMRI found that schizophrenia risk was linked to increased DMN and CEN connectivity and reduced attention network connectivity (Mu, Dang, & Luo, 2024). These findings are significant for NIBS, not only for treatment but also for preventative targets for at-risk individuals and may enable earlier interventions to modify the course of psychiatric disorders.

Inclusion of pre-treatment network physiological properties in NIBS studies is an important factor for treatment response. For example, pre-TMS neural activity predicts post-TMS responses (Pasley, Allen, & Freeman, 2009), supporting its use for patient stratification and treatment optimisation. Pre-NIBS treatment measures include EEG, fMRI for activity levels, rsfMRI for connectivity strength, and TMS measures of cortical E/I. One such example is SICI, a marker of cortical GABA-A inhibition, which is reliably reduced in SCZ and may enable disorder-specific and treatment biomarkers (Lányi et al., 2024).

Variations in the stimulation parameters, including intensity, number of pulses, sham procedures for rTMS (Li et al., 2020), and number of sessions and frequency of stimulation for tDCS (Yang et al., 2019) may affect their therapeutic efficacy and highlight the need for refinement and standardization of treatment protocols.

NIBS offers a promising therapeutic strategy, either alone or in combination with existing therapeutic approaches for psychiatric disorders and symptoms that fail to respond to conventional treatments. Large-scale, RCTs with long-term follow-up are essential to establish optimal protocols and evaluate safety comprehensively. Efforts should also be directed towards the development of more practical and accessible treatment systems and training programs to facilitate more widespread clinical use. While challenges remain, ongoing research is bringing NIBS closer to becoming a mainstream, patient-centered, mechanism-based treatment for psychiatric disorders and potentially offering earlier interventions that could modify the course of psychiatric disorders.

## Figures and Tables

**Table 1:** Major brain networks involved in psychiatric disorders: Core nodes and key functions Major brain networks Major nodes Key functions

Major brain networks	Major nodes	Key functions
**Default Mode Network (DMN)** (Menon, 2023; Smith et al., 2009 ; Yeo et al., 2011)	PCC mPFC Precuneus Angular Gyrus	More active at rest than active task engagement Introspection, internal narrative, self-reflection Mind wandering and spontaneous cognition Autobiographical memory retrieval and prospective memory Theory of mind and mentalizing
**Central Executive Network (CEN) / Frontoparietal Network (FPN)** (Smith et al., 2009; Yeo et al., 2011)	Bilateral DLPFC Posterior Parietal Cortex Intraparietal Sulcus	Executive functions, including working memory, cognitive control, decision-making, problem-solving, and cognitive resource allocation to goal-directed behaviors ‘Anticorrelated’ with the DMN and works in concert with the SN
**Salience Network (SN)** (Schimmelpfennig, Topczewski, Zajkowski, & Jankowiak-Siuda, 2023; Uddin, 2015)	ACC Anterior Insula vmPFC Amygdala Substantia Nigra	Salient stimuli identification (bottom-up) and arousal (top-down) allocation Switching between DMN & CEN Emotional processing Regulation of autonomic responses
**Affective Network (AN)** (Zeng et al., 2012)	OFC sgACC Limbic structures (amygdala, hippocampus, insula)	Emotion generation and assigning emotional valence to stimuli Emotional processing and regulation
**Reward Network (RN)** (Haber, 2009)	Frontal cortex ACC Dorsal striatum (caudate and putamen) Ventral striatum (NAc) VTA Limbic structures (amygdala, hippocampus)	Ventral striatum - Reward-related pleasure generation - Motivation and incentive salience - Reinforcement learning Fronto-basal RN - Reward value and reward-related risks evaluation - Reward prediction and anticipation - Decision-making on reward/risk evaluation, action planning
**Dorsal Attention Network (DAN)** (Corbetta & Shulman, 2002)	FEF Intraparietal Sulcus Superior Parietal Lobule MT+	Voluntary, top-down attention Goal-directed voluntary control of visuospatial attention
**Ventral Attention Network (VAN)** (Corbetta & Shulman, 2002)	TPJ Inferior Frontal Gyrus Ventral Frontal Cortex Middle Frontal Gyrus	Involuntary, bottom-up attention Unattended/unexpected stimuli detection and attention shifting VAN and DAN interaction switches attention between top-down goals and bottom-up stimuli
**Limbic Network** (Yeo et al., 2011)	Amygdala Hippocampus OFC Cingulate Cortex Hypothalamus NAc	Emotional processing Motivation Memory encoding and retrieval Visceromotor regulation
**Sensorimotor Network (SMN)** (Uddin, Yeo, & Spreng, 2019; Xiong, Tian, Zeng, Huang, & Wang, 2021)	M1 S1 Premotor Cortex SMA Middle cingulate cortex Basal Ganglia – Thal- amus Cerebellum	Processing sensory inputs and motor outputs Motor coordination Proprioception Sensory Integration
**Visual Network (VN)** (Xiong et al., 2021)	V1 LGN Visual Association Areas (V2, V3, V4, V5/MT) Occipital Lobe	Visual perception, object recognition, spatial awareness
**Auditory network** (Kuiper et al., 2020)	A1 in Heschl’s Gyrus A2 STG MGN Insula IFG	Sound localization and discrimination, language processing

Abbreviations: PCC: Posterior Cingulate Cortex; mPFC: medial Prefrontal Cortex;; DLPFC: Dorsolateral Prefrontal Cortex; dAIC: dorsal Anterior Insular Cortex; ACC: Anterior Cingulate Cortex; vmPFC: Ventromedial Prefrontal Cortex; OFC: Orbitofrontal Cortex; sgACC: subgenual Anterior Cingulate Cortex; VTA: Ventral Tegmental Area; NAc: Nucleus accumbens; FEF: Frontal Eye Fields; MT+: Middle Temporal Motion Complex; TPJ: Temporoparietal Junction; M1: Primary Motor Cortex; S1: Primary Somatosensory Cortex; SMA: Supplementary Motor Area; V1: Primary Visual Cortex; LGN: Lateral Geniculate Nucleus (thalamus); A1: Primary Auditory Cortex; A2: Auditory Association Cortex; STG: Superior Temporal Gyrus; MGN: Medial Geniculate Body (thalamus); IFG: Inferior Frontal Gyrus.

## References

[R1] Abramovitch A, Abramowitz JS, Mittelman A (2013). The neuropsychology of adult obsessive–compulsive disorder: A meta-analysis. Clinical Psychology Review.

[R2] Abramovitch A, Short T, Schweiger A (2021). The C factor: Cognitive dysfunction as a transdiagnostic dimension in psychopathology. Clinical Psychology Review.

[R3] Aleman A, Enriquez-Geppert S, Knegtering H, Dlabac-de Lange JJ (2018). Moderate effects of noninvasive brain stimulation of the frontal cortex for improving negative symptoms in schizophrenia: Meta-analysis of controlled trials. Neuroscience & Biobehavioral Reviews.

[R4] Alexander GE, Crutcher MD (1990). Functional architecture of basal ganglia circuits: Neural substrates of parallel processing. Trends in Neurosciences.

[R5] Anticevic A, Haut K, Murray JD, Repovs G, Yang GJ, Diehl C, McEwen SC, Bearden CE, Addington J, Goodyear B, Cadenhead KS (2015). Association of Thalamic dysconnectivity and conversion to psychosis in youth and Young adults at elevated clinical risk. JAMA Psychiatry.

[R6] Appelbaum LG, Daniels H, Lochhead L, Bacio B, Cash R, Weissman CR, Kohn JN, Hadas I, Daskalakis ZJ (2025). Accelerated intermittent theta-burst stimulation for treatment-resistant bipolar depression: A randomized clinical trial. JAMA Network Open.

[R7] Avram M, Brandl F, Bäuml J, Sorg C (2018). Cortico-thalamic hypo- and hyperconnectivity extend consistently to basal ganglia in schizophrenia. Neuropsychopharmacology.

[R8] Bailey NW, Hoy KE, Rogasch NC, Thomson RH, McQueen S, Elliot D, Sullivan CM, Fulcher BD, Daskalakis ZJ, Fitzgerald PB (2018). Responders to rTMS for depression show increased fronto-midline theta and theta connectivity compared to non-responders. Brain Stimulation.

[R9] Begemann MJ, Brand BA, Curcic-Blake B, Aleman A, Sommer IE (2020). Efficacy of non-invasive brain stimulation on cognitive functioning in brain disorders: A meta-analysis. Psychological Medicine.

[R10] Brady RO, Gonsalvez I, Lee I, Öngür D, Seidman LJ, Schmahmann JD, Eack SM, Keshavan MS, Pascual-Leone A, Halko MA (2019). Cerebellar-prefrontal network connectivity and negative symptoms in schizophrenia. American Journal of Psychiatry.

[R11] Brody AL, Saxena S, Mandelkern MA, Fairbanks LA, Ho ML, Baxter LR (2001). Brain metabolic changes associated with symptom factor improvement in major depressive disorder. Biological Psychiatry.

[R12] Bruder GE, Stewart JW, McGrath PJ (2017). Right brain, left brain in depressive disorders: Clinical and theoretical implications of behavioral, electrophysiological and neuroimaging findings. Neuroscience & Biobehavioral Reviews.

[R13] Bruin WB, Abe Y, Alonso P, Anticevic A, Backhausen LL, Balachander S, Bargallo N, Batistuzzo MC, Benedetti F, Bertolin Triquell S, Brem S (2023). The functional connectome in obsessive-compulsive disorder: Resting-state mega-analysis and machine learning classification for the ENIGMA-OCD consortium. Molecular Psychiatry.

[R14] Brunoni AR, Chaimani A, Moffa AH, Razza LB, Gattaz WF, Daskalakis ZJ, Carvalho AF (2017). Repetitive transcranial magnetic stimulation for the acute treatment of major depressive episodes: A systematic review with network meta-analysis. JAMA Psychiatry.

[R15] Brunoni AR, Moffa AH, Fregni F, Palm U, Padberg F, Blumberger DM, Daskalakis ZJ, Bennabi D, Haffen E, Alonzo A, Loo CK (2016). Transcranial direct current stimulation for acute major depressive episodes: Meta-analysis of individual patient data. British Journal of Psychiatry.

[R16] Bulubas L, Padberg F, Bueno PV, Duran F, Busatto G, Amaro E, Benseñor IM, Lotufo PA, Goerigk S, Gattaz W, Keeser D (2019). Antidepressant effects of tDCS are associated with prefrontal gray matter volumes at baseline: Evidence from the ELECT-TDCS trial. Brain Stimulation.

[R17] Burton CZ, Garnett EO, Capellari E, Chang S-E, Tso IF, Hampstead M, Taylor SF (2023). Combined cognitive training and transcranial direct current stimulation in neuropsychiatric disorders: A systematic review and meta-analysis. Biological Psychiatry: Cognitive Neuroscience and Neuroimaging.

[R18] Cardoso F, McHayle Z (2024). The economic and social costs of mental ill health: Review of methodology and update of calculations.

[R19] Cash RFH, Cocchi L, Lv J, Wu Y, Fitzgerald PB, Zalesky A (2021). Personalized connectivity-guided DLPFC-TMS for depression: Advancing computational feasibility, precision and reproducibility. Human Brain Mapping.

[R20] Celada P, Puig MV, Artigas F (2013). Serotonin modulation of cortical neurons and networks. Frontiers in Integrative Neuroscience.

[R21] Chail A, Saini R, Bhat P, Srivastava K, Chauhan V (2018). Transcranial magnetic stimulation: A review of its evolution and current applications. Industrial Psychiatry Journal.

[R22] Chavez-Baldini U, Nieman DH, Keestra A, Lok A, Mocking RJT, de Koning P, Krzhizhanovskaya VV, Bockting CLH, van Rooijen G, Smit DJA, Sutterland AL (2023). The relationship between cognitive functioning and psychopathology in patients with psychiatric disorders: A transdiagnostic network analysis. Psychological Medicine.

[R23] Cheng J-L, Tan C, Liu H-Y, Han D-M, Liu Z-C (2023). Past, present, and future of deep transcranial magnetic stimulation: A review in psychiatric and neurological disorders. World Journal of Psychiatry.

[R24] Corbetta M, Shulman GL (2002). Control of goal-directed and stimulus-driven attention in the brain. Nature Reviews Neuroscience.

[R25] Cox SS, Connolly DJ, Peng X, Badran BW (2025). A comprehensive review of low-intensity focused ultrasound parameters and applications in neurologic and psychiatric disorders. Neuromodulation: Technology at the neural Interface.

[R26] Davey CG, Harrison BJ, Yücel M, Allen NB (2012). Regionally specific alterations in functional connectivity of the anterior cingulate cortex in major depressive disorder. Psychological Medicine.

[R27] Davey CG, Whittle S, Harrison BJ, Simmons JG, Byrne ML, Schwartz OS, Allen NB (2015). Functional brain-imaging correlates of negative affectivity and the onset of first-episode depression. Psychological Medicine.

[R28] De Wit SJ, De Vries FE, Van Der Werf YD, Cath DC, Heslenfeld DJ, Veltman EM, Van Balkom AJLM, Veltman DJ, Van Den Heuvel OA (2012). Presupplementary motor area hyperactivity during response inhibition: A candidate Endophenotype of obsessive-compulsive disorder. American Journal of Psychiatry.

[R29] Di Hou M, Santoro V, Biondi A, Shergill SS, Premoli I (2021). A systematic review of TMS and neurophysiological biometrics in patients with schizophrenia. Journal of Psychiatry and Neuroscience.

[R30] Dinur-Klein L, Dannon P, Hadar A, Rosenberg O, Roth Y, Kotler M, Zangen A (2014). Smoking cessation induced by deep repetitive transcranial magnetic stimulation of the prefrontal and insular cortices: A prospective. Randomized Controlled Trial Biological Psychiatry.

[R31] Downar J, Blumberger DM, Daskalakis ZJ (2016). The neural crossroads of psychiatric illness: An emerging target for brain stimulation. Trends in Cognitive Sciences.

[R32] Echevarria MAN, Batistuzzo MC, Silva RMF, Brunoni AR, Sato JR, Miguel EC, Hoexter MQ, Shavitt RG (2024). Increases in functional connectivity between the default mode network and sensorimotor network correlate with symptomatic improvement after transcranial direct current stimulation for obsessive-compulsive disorder. Journal of Affective Disorders.

[R33] Figee M, Vink M, De Geus F, Vulink N, Veltman DJ, Westenberg H, Denys D (2011). Dysfunctional reward circuitry in obsessive-compulsive disorder. Biological Psychiatry.

[R34] Fitzgerald PB, Hoy K, McQueen S, Herring S, Segrave R, Been G, Kulkarni J, Daskalakis ZJ (2008). Priming stimulation enhances the effectiveness of low-frequency right prefrontal cortex transcranial magnetic stimulation in major depression. Journal of Clinical Psychopharmacology.

[R35] Fitzsimmons SMDD, Oostra E, Postma TS, Van Der Werf YD, Van Den Heuvel OA (2024). Repetitive transcranial magnetic stimulation–induced neuroplasticity and the treatment of psychiatric disorders: State of the evidence and future opportunities. Biological Psychiatry.

[R36] Fox MD, Buckner RL, White MP, Greicius MD, Pascual-Leone A (2012). Efficacy of transcranial magnetic stimulation targets for depression is related to intrinsic functional connectivity with the Subgenual cingulate. Biological Psychiatry.

[R37] Friston K, Brown HR, Siemerkus J, Stephan KE (2016). The dysconnection hypothesis (2016). Schizophrenia Research.

[R38] Friston KJ, Frith CD (1995). Schizophrenia: A disconnection syndrome?. Clinical Neuroscience.

[R39] Fusar-Poli P, Papanastasiou E, Stahl D, Rocchetti M, Carpenter W, Shergill S, McGuire P (2015). Treatments of negative symptoms in schizophrenia: Meta-analysis of 168 randomized placebo-controlled trials. Schizophrenia Bulletin.

[R40] Gefferie SR, Jiménez-Jiménez D, Visser GH, Helling RM, Sander JW, Balestrini S, Thijs RD (2023). Transcranial magnetic stimulation-evoked electroencephalography responses as biomarkers for epilepsy: A review of study design and outcomes. Human Brain Mapping.

[R41] Giordano GM, Stanziano M, Papa M, Mucci A, Prinster A, Soricelli A, Galderisi S (2018). Functional connectivity of the ventral tegmental area and avolition in subjects with schizophrenia: A resting state functional MRI study. European Neuropsychopharmacology.

[R42] Goghari VM, Sponheim SR, MacDonald AW (2010). The functional neuroanatomy of symptom dimensions in schizophrenia: A qualitative and quantitative review of a persistent question. Neuroscience & Biobehavioral Reviews.

[R43] Gonsalves MA, White TL, Barredo J, DeMayo MM, DeLuca E, Harris AD, Carpenter LL (2024). Cortical glutamate, Glx, and total N-acetylaspartate: Potential biomarkers of repetitive transcranial magnetic stimulation treatment response and outcomes in major depression. Translational Psychiatry.

[R44] Grassi G, Moradei C, Cecchelli C (2023). Will transcranial magnetic stimulation improve the treatment of obsessive–compulsive disorder? A systematic review and meta-analysis of current targets and clinical evidence. Life.

[R45] Gromann PM, Tracy DK, Giampietro V, Brammer MJ, Krabbendam L, Shergill SS (2012). Examining frontotemporal connectivity and rTMS in healthy controls: Implications for auditory hallucinations in schizophrenia. Neuropsychology.

[R46] Grützmann R, Klawohn J, Elsner B, Reuter B, Kaufmann C, Riesel A, Bey K, Heinzel S, Kathmann N (2022). Error-related activity of the sensorimotor network contributes to the prediction of response to cognitive-behavioral therapy in obsessive–compulsive disorder. NeuroImage: Clinical.

[R47] Haber SN, Dreher J-C, Tremblay L (2009). Handbook of reward and decision making.

[R48] Hadas I, Sun Y, Lioumis P, Zomorrodi R, Jones B, Voineskos D, Downar J, Fitzgerald PB, Blumberger DM, Daskalakis ZJ (2019). Association of repetitive transcranial magnetic stimulation treatment with subgenual cingulate hyperactivity in patients with major depressive disorder: A secondary analysis of a randomized clinical trial. JAMA Network Open.

[R49] He H, Lu J, Yang L, Zheng J, Gao F, Zhai Y, Feng J, Fan Y, Ma X (2017). Repetitive transcranial magnetic stimulation for treating the symptoms of schizophrenia: A PRISMA compliant meta-analysis. Clinical Neurophysiology.

[R50] Herrmann CS, Rach S, Neuling T, Strüber D (2013). Transcranial alternating current stimulation: A review of the underlying mechanisms and modulation of cognitive processes. Frontiers in Human Neuroscience.

[R51] Homan P, Kindler J, Hauf M, Hubl D, Dierks T (2012). Cerebral blood flow identifies responders to transcranial magnetic stimulation in auditory verbal hallucinations. Translational Psychiatry.

[R52] Horne CM, Sahni A, Pang SW, Vanes LD, Szentgyorgyi T, Averbeck B, Moran RJ, Shergill SS (2022). The role of cognitive control in the positive symptoms of psychosis. NeuroImage: Clinical.

[R53] Horne CM, Vanes LD, Verneuil T, Mouchlianitis E, Szentgyorgyi T, Averbeck B, Leech R, Moran RJ, Shergill SS (2021). Cognitive control network connectivity differentially disrupted in treatment resistant schizophrenia. NeuroImage: Clinical.

[R54] Howes OD, Shatalina E (2022). Integrating the neurodevelopmental and dopamine hypotheses of schizophrenia and the role of cortical excitation-inhibition balance. Biological Psychiatry.

[R55] Howes OD, Thase ME, Pillinger T (2022). Treatment resistance in psychiatry: State of the art and new directions. Molecular Psychiatry.

[R56] Hyde J, Carr H, Kelley N, Seneviratne R, Reed C, Parlatini V, Garner M, Solmi M, Rosson S, Cortese S, Brandt V (2022). Efficacy of neurostimulation across mental disorders: Systematic review and meta-analysis of 208 randomized controlled trials. Molecular Psychiatry.

[R57] Ishida T, Nakamura Y, Tanaka SC, Mitsuyama Y, Yokoyama S, Shinzato H, Itai E, Okada G, Kobayashi Y, Kawashima T, Miyata J (2023). Aberrant large-scale network interactions across psychiatric disorders revealed by large-sample multi-site resting-state functional magnetic resonance imaging datasets. Schizophrenia Bulletin.

[R58] Jiang Y, Guo Z, Xing G, He L, Peng H, Du F, McClure MA, Mu Q (2019). Effects of high-frequency transcranial magnetic stimulation for cognitive deficit in schizophrenia: A meta-analysis. Frontiers in Psychiatry.

[R59] Kaiser RH, Andrews-Hanna JR, Wager TD, Pizzagalli DA (2015). Large-scale network dysfunction in major depressive disorder: A meta-analysis of resting-state functional connectivity. JAMA Psychiatry.

[R60] Kar SK, Agrawal A, Silva-dos-Santos A, Gupta Y, Deng Z-D (2024). The efficacy of transcranial magnetic stimulation in the treatment of obsessive-compulsive disorder: An umbrella review of meta-analyses. CNS Spectrums.

[R61] Kennedy NI, Lee WH, Frangou S (2018). Efficacy of non-invasive brain stimulation on the symptom dimensions of schizophrenia: A meta-analysis of randomized controlled trials. European Psychiatry.

[R62] Kesikburun S (2022). Non-invasive brain stimulation in rehabilitation. Turkish Journal of Physical Medicine and Rehabilitation.

[R63] Koenigs M, Grafman J (2009). The functional neuroanatomy of depression: Distinct roles for ventromedial and dorsolateral prefrontal cortex. Behavioural Brain Research.

[R64] Koutsouleris N, Wobrock T, Guse B, Langguth B, Landgrebe M, Eichhammer P, Frank E, Cordes J, Wölwer W, Musso F, Winterer G (2018). Predicting response to repetitive transcranial magnetic stimulation in patients with schizophrenia using structural magnetic resonance imaging: A multisite machine learning analysis. Schizophrenia Bulletin.

[R65] Kuiper JJ, Lin Y-H, Young IM, Bai MY, Briggs RG, Tanglay O, Fonseka RD, Hormovas J, Dhanaraj V, Conner AK, O’Neal CM (2020). A parcellation-based model of the auditory network. Hearing Research.

[R66] Lányi O, Koleszár B, Schulze Wenning A, Balogh D, Engh MA, Horváth AA, Fehérvari P, Hegyi P, Molnár Z, Unoka Z, Csukly G (2024). Excitation/inhibition imbalance in schizophrenia: A meta-analysis of inhibitory and excitatory TMS-EMG paradigms. Schizophrenia.

[R67] Leichsenring F, Steinert C, Rabung S, Ioannidis JPA (2022). The efficacy of psychotherapies and pharmacotherapies for mental disorders in adults: An umbrella review and meta-analytic evaluation of recent meta-analyses. World Psychiatry.

[R68] Li BJ, Friston K, Mody M, Wang HN, Lu HB, Hu DW (2018). A brain network model for depression: From symptom understanding to disease intervention. CNS Neuroscience & Therapeutics.

[R69] Li C, Hsieh J, Huang H, Chen MH, Juan CH, Tu PC, Lee YC, Wang SJ, Cheng CM, Su T-P (2016). Cognition-modulated frontal activity in prediction and augmentation of antidepressant efficacy: A randomized controlled pilot study. Cerebral Cortex.

[R70] Li J, Cao X, Liu S, Li X, Xu Y (2020). Efficacy of repetitive transcranial magnetic stimulation on auditory hallucinations in schizophrenia: A meta-analysis. Psychiatry Research.

[R71] Liddle PF (1987). Schizophrenic syndromes, cognitive performance and neurological dysfunction. Psychological Medicine.

[R72] Liu J, Cao L, Li H, Gao Y, Bu X, Liang K, Bao W, Zhang S, Qiu H, Li X, Hu X (2022). Abnormal resting-state functional connectivity in patients with obsessive-compulsive disorder: A systematic review and meta-analysis. Neuroscience & Biobehavioral Reviews.

[R73] Lorentzen R, Nguyen TD, McGirr A, Hieronymus F, Østergaard SD (2022). The efficacy of transcranial magnetic stimulation (TMS) for negative symptoms in schizophrenia: A systematic review and meta-analysis. Schizophrenia.

[R74] Lynch CJ, Elbau IG, Ng T, Ayaz A, Zhu S, Wolk D, Manfredi N, Johnson M, Chang M, Chou J, Summerville I (2024). Frontostriatal salience network expansion in individuals in depression. Nature.

[R75] Mayberg HS (2003). Modulating dysfunctional limbic-cortical circuits in depression: Towards development of brain-based algorithms for diagnosis and optimised treatment. British Medical Bulletin.

[R76] Mayberg HS, Liotti M, Brannan SK, McGinnis S, Mahurin RK, Jerabek PA, Silva JA, Tekell JL, Martin CC, Lancaster JL, Fox PT (1999). Reciprocal limbic-cortical function and negative mood: Converging PET findings in depression and Normal sadness. American Journal of Psychiatry.

[R77] McGirr A, Vila-Rodriguez F, Cole J, Torres IJ, Arumugham SS, Keramatian K, Saraf G, Lam RW, Chakrabarty T, Yatham LN (2021). Efficacy of active vs sham intermittent theta burst transcranial magnetic stimulation for patients with bipolar depression: A randomized clinical trial. JAMA Network Open.

[R78] McGovern RA, Sheth SA (2017). Role of the dorsal anterior cingulate cortex in obsessive-compulsive disorder: Converging evidence from cognitive neuroscience and psychiatric neurosurgery. Journal of Neurosurgery.

[R79] McIntyre RS, Alsuwaidan M, Baune BT, Berk M, Demyttenaere K, Goldberg JF, Gorwood P, Ho R, Kasper S, Kennedy SH, Ly-Uson J (2023). Treatment-resistant depression: definition, prevalence, detection, management, and investigational interventions. World Psychiatry.

[R80] McTeague LM, Goodkind MS, Etkin A (2016). Transdiagnostic impairment of cognitive control in mental illness. Journal of Psychiatric Research.

[R81] McTeague LM, Huemer J, Carreon DM, Jiang Y, Eickhoff SB, Etkin A (2017). Identification of common neural circuit disruptions in cognitive control across psychiatric disorders. American Journal of Psychiatry.

[R82] Menon V (2011). Large-scale brain networks and psychopathology: A unifying triple network model. Trends in Cognitive Sciences.

[R83] Menon V (2020). Brain networks and cognitive impairment in psychiatric disorders. World Psychiatry.

[R84] Menon V (2023). 20 years of the default mode network: A review and synthesis. Neuron.

[R85] Mu C, Dang X, Luo X-J (2024). Mendelian randomization analyses reveal causal relationships between brain functional networks and risk of psychiatric disorders. Nature Human Behaviour.

[R86] Murray RM, Bhavsar V, Tripoli G, Howes O (2017). 30 years on: How the neurodevelopmental hypothesis of schizophrenia morphed into the developmental risk factor model of psychosis. Schizophrenia Bulletin.

[R87] Mutz J, Vipulananthan V, Carter B, Hurlemann R, Fu CHY, Young AH (2019). Comparative efficacy and acceptability of non-surgical brain stimulation for the acute treatment of major depressive episodes in adults: Systematic review and network meta-analysis. BMJ.

[R88] National Institute for Health and Care Excellence (2015). Repetitive transcranial magnetic stimulation for depression: Interventional procedures guidance. Behavioural and Neurodevelopmental Conditions: Mental Health.

[R89] National Institute for Health and Care Excellence (2020). Transcranial magnetic stimulation for obsessive-compulsive disorder: Interventional procedures guidance. Behavioural and Neurodevelopmental Conditions: Mental Health.

[R90] National Institute for Health and Care Excellence (2024). Obsessive-compulsive disorder.

[R91] Ng TH, Alloy LB, Smith DV (2019). Meta-analysis of reward processing in major depressive disorder reveals distinct abnormalities within the reward circuit. Translational Psychiatry.

[R92] Nguyen K-H, Gordon LG (2015). Cost-effectiveness of repetitive transcranial magnetic stimulation versus antidepressant therapy for treatment-resistant depression. Value in Health.

[R93] Nord CL, Halahakoon DC, Limbachya T, Charpentier C, Lally N, Walsh V, Leibowitz J, Pilling S, Roiser JP (2019). Neural predictors of treatment response to brain stimulation and psychological therapy in depression: A double-blind randomized controlled trial. Neuropsychopharmacology.

[R94] Norman LJ, Carlisi CO, Christakou A, Murphy CM, Chantiluke K, Giampietro V, Simmons A, Brammer M, Mataix-Cols D, Rubia K (2018). Frontostriatal dysfunction during decision making in attention-deficit/hyperactivity disorder and obsessive-compulsive disorder. Biological Psychiatry: Cognitive Neuroscience and Neuroimaging.

[R95] Orlov ND, Muqtadir SA, Oroojeni H, Averbeck B, Rothwell J, Shergill SS (2022). Stimulating learning: A functional MRI and behavioral investigation of the effects of transcranial direct current stimulation on stochastic learning in schizophrenia. Psychiatry Research.

[R96] Orlov ND, O’Daly O, Tracy DK, Daniju Y, Hodsoll J, Valdearenas L, Rothwell J, Shergill SS (2017). Stimulating thought: A functional MRI study of transcranial direct current stimulation in schizophrenia. Brain.

[R97] Orlov ND, Tracy DK, Joyce D, Patel S, Rodzinka-Pasko J, Dolan H, Hodsoll J, Collier T, Rothwell J, Shergill SS (2017). Stimulating cognition in schizophrenia: A controlled pilot study of the effects of pre-frontal transcranial direct current stimulation upon memory and learning. Brain Stimulation.

[R98] Otani VHO, Shiozawa P, Cordeiro Q, Uchida RR (2015). A systematic review and meta-analysis of the use of repetitive transcranial magnetic stimulation for auditory hallucinations treatment in refractory schizophrenic patients. International Journal of Psychiatry in Clinical Practice.

[R99] Pascual-Leone A, Valls-Solé J, Wassermann EM, Hallett M (1994). Responses to rapid-rate transcranial magnetic stimulation of the human motor cortex. Brain.

[R100] Pasley BN, Allen EA, Freeman RD (2009). State-dependent variability of neuronal responses to transcranial magnetic stimulation of the visual cortex. Neuron.

[R101] Paul AK, Bose A, Kalmady SV, Shivakumar V, Sreeraj VS, Parlikar R, Narayanaswamy JC, Dursun SM, Greenshaw AJ, Greiner R, Venkatasubramanian G (2022). Superior temporal gyrus functional connectivity predicts transcranial direct current stimulation response in schizophrenia: A machine learning study. Frontiers in Psychiatry.

[R102] Perera MPN, Gotsis ES, Bailey NW, Fitzgibbon BM, Fitzgerald PB (2024). Exploring functional connectivity in large-scale brain networks in obsessive-compulsive disorder: A systematic review of EEG and fMRI studies. Cerebral Cortex.

[R103] Pettersson-Yeo W, Allen P, Benetti S, McGuire P, Mechelli A (2011). Dysconnectivity in schizophrenia: Where are we now?. Neuroscience & Biobehavioral Reviews.

[R104] Pinto BS, Cavendish BA, Da Silva PHR, Suen PJC, Marinho KAP, Valiengo LDCL, Vanderhasselt M-A, Brunoni AR, Razza LB (2022). The effects of transcranial direct current stimulation in obsessive–compulsive disorder symptoms: A meta-analysis and integrated electric fields modeling analysis. Biomedicine.

[R105] Rabinowitz J, Levine SZ, Garibaldi G, Bugarski-Kirola D, Berardo CG, Kapur S (2012). Negative symptoms have greater impact on functioning than positive symptoms in schizophrenia: Analysis of CATIE data. Schizophrenia Research.

[R106] Riesel A, Klawohn J, Grützmann R, Kaufmann C, Heinzel S, Bey K, Lennertz L, Wagner M, Kathmann N (2019). Error-related brain activity as a transdiagnostic endophenotype for obsessive-compulsive disorder, anxiety and substance use disorder. Psychological Medicine.

[R107] Santoro V, Hou MD, Premoli I, Belardinelli P, Biondi A, Carobin A, Puledda F, Michalopoulou PG, Richardson MP, Rocchi L, Shergill SS (2024). Investigating cortical excitability and inhibition in patients with schizophrenia: A TMS-EEG study. Brain Research Bulletin.

[R108] Schimmelpfennig J, Topczewski J, Zajkowski W, Jankowiak-Siuda K (2023). The role of the salience network in cognitive and affective deficits. Frontiers in Human Neuroscience.

[R109] Schrammen E, Roesmann K, Rosenbaum D, Redlich R, Harenbrock J, Dannlowski U, Leehr EJ (2022). Functional neural changes associated with psychotherapy in anxiety disorders – A meta-analysis of longitudinal fMRI studies. Neuroscience & Biobehavioral Reviews.

[R110] Segal A, Parkes L, Aquino K, Kia SM, Wolfers T, Franke B, Hoogman M, Beckmann CF, Westlye LT, Andreassen OA, Zalesky A (2023). Regional, circuit and network heterogeneity of brain abnormalities in psychiatric disorders. Nature Neuroscience.

[R111] Sha Z, Wager TD, Mechelli A, He Y (2019). Common dysfunction of large-scale neurocognitive networks across psychiatric disorders. Biological Psychiatry.

[R112] Shao X, Liao Y, Gu L, Chen W, Tang J (2021). The Etiology of auditory hallucinations in schizophrenia: From multidimensional levels. Frontiers in Neuroscience.

[R113] Sheffield JM, Karcher NR, Barch DM (2018). Cognitive deficits in psychotic disorders: A lifespan perspective. Neuropsychology Review.

[R114] Shephard E, Stern ER, Van Den Heuvel OA, Costa DLC, Batistuzzo MC, Godoy PBG, Lopes AC, Brunoni AR, Hoexter MQ, Shavitt RG, Reddy YCJ (2021). Toward a neurocircuit-based taxonomy to guide treatment of obsessive–compulsive disorder. Molecular Psychiatry.

[R115] Shergill SS, Brammer MJ, Williams SCR, Murray RM, McGuire PK (2000). Mapping auditory hallucinations in schizophrenia using functional magnetic resonance imaging. Archives of General Psychiatry.

[R116] Shi R, Wang Z, Yang D, Hu Y, Zhang Z, Lan D, Su Y, Wang Y (2024). Short-term and long-term efficacy of accelerated transcranial magnetic stimulation for depression: A systematic review and meta-analysis. BMC Psychiatry.

[R117] Shi Y, Wu W (2025). Advances in transcranial focused ultrasound neuromodulation for mental disorders. Progress in Neuro-Psychopharmacology and Biological Psychiatry.

[R118] Siddiqi SH, Schaper FLWVJ, Horn A, Hsu J, Padmanabhan JL, Brodtmann A, Cash RFH, Corbetta M, Choi KS, Dougherty DD, Egorova N (2021). Brain stimulation and brain lesions converge on common causal circuits in neuropsychiatric disease. Nature Human Behaviour.

[R119] Slotema CW, Aleman A, Daskalakis ZJ, Sommer IE (2012). Meta-analysis of repetitive transcranial magnetic stimulation in the treatment of auditory verbal hallucinations: Update and effects after one month. Schizophrenia Research.

[R120] Slotema CW, Blom JD, Van Lutterveld R, Hoek HW, Sommer IEC (2014). Review of the efficacy of transcranial magnetic stimulation for auditory verbal hallucinations. Biological Psychiatry.

[R121] Smith SM, Fox PT, Miller KL, Glahn DC, Fox PM, Mackay CE, Filippini N, Watkins KE, Toro R, Laird AR, Beckmann CF (2009). Correspondence of the brain’s functional architecture during activation and rest. Proceedings of the National Academy of Sciences.

[R122] Sporns O (2014). Contributions and challenges for network models in cognitive neuroscience. Nature Neuroscience.

[R123] Stuchlíková Z, Klírová M (2022). A literature mini-review of transcranial direct current stimulation in schizophrenia. Frontiers in Psychiatry.

[R124] Suhas S, Malo PK, Kumar V, Issac TG, Chithra NK, Bhaskarapillai B, Reddy YCJ, Rao NP (2023). Treatment strategies for serotonin reuptake inhibitor-resistant obsessive-compulsive disorder: A network meta-analysis of randomised controlled trials. The World Journal of Biological Psychiatry.

[R125] Tan X, Goh SE, Lee JJ, Vanniasingham SD, Brunelin J, Lee J, Tor PC (2023). Efficacy of using intermittent theta burst stimulation to treat negative symptoms in patients with schizophrenia—A systematic review and meta-analysis. Brain Sciences.

[R126] Tee MMK, Au CH (2020). A systematic review and meta-analysis of randomized sham-controlled trials of repetitive transcranial magnetic stimulation for bipolar disorder. Psychiatric Quarterly.

[R127] Thorsen AL, Hagland P, Radua J, Mataix-Cols D, Kvale G, Hansen B, Van Den Heuvel OA (2018). Emotional processing in obsessive-compulsive disorder: A systematic review and meta-analysis of 25 functional neuroimaging studies. Biological Psychiatry: Cognitive Neuroscience and Neuroimaging.

[R128] Tomasi D, Shokri-Kojori E, Volkow ND (2016). High-resolution functional connectivity density: Hub locations, sensitivity, specificity, reproducibility, and reliability. Cerebral Cortex.

[R129] Turrigiano GG, Nelson SB (2004). Homeostatic plasticity in the developing nervous system. Nature Reviews Neuroscience.

[R130] Uddin LQ (2015). Salience processing and insular cortical function and dysfunction. Nature Reviews Neuroscience.

[R131] Uddin LQ, Yeo BTT, Spreng RN (2019). Towards a universal taxonomy of macro-scale functional human brain networks. Brain Topography.

[R132] Valiengo LDCL, Goerigk S, Gordon PC, Padberg F, Serpa MH, Koebe S, Santos LAD, Lovera RAM, Carvalho JBD, Van De Bilt M, Lacerda ALT (2020). Efficacy and safety of transcranial direct current stimulation for treating negative symptoms in schizophrenia: A randomized clinical trial. JAMA Psychiatry.

[R133] Van Den Heuvel OA, Van Wingen G, Soriano-Mas C, Alonso P, Chamberlain SR, Nakamae T, Denys D, Goudriaan AE, Veltman DJ (2016). Brain circuitry of compulsivity. European Neuropsychopharmacology.

[R134] Vida RG, Sághy E, Bella R, Kovács S, Erdősi D, Józwiak-Hagymásy J, Zemplényi A, Tényi T, Osváth P, Voros V (2023). Efficacy of repetitive transcranial magnetic stimulation (rTMS) adjunctive therapy for major depressive disorder (MDD) after two antidepressant treatment failures: Meta-analysis of randomized sham-controlled trials. BMC Psychiatry.

[R135] Vigo D, Thornicroft G, Atun R (2016). Estimating the true global burden of mental illness. The Lancet Psychiatry.

[R136] Voetterl HTS, Sack AT, Olbrich S, Stuiver S, Rouwhorst R, Prentice A, Pizzagalli DA, Van Der Vinne N, Van Waarde JA, Brunovsky M, Van Oostrom I (2023). Alpha peak frequency-based Brainmarker-I as a method to stratify to pharmacotherapy and brain stimulation treatments in depression. Nature Mental Health.

[R137] Voigt J, Carpenter L, Leuchter A (2017). Cost effectiveness analysis comparing repetitive transcranial magnetic stimulation to antidepressant medications after a first treatment failure for major depressive disorder in newly diagnosed patients – A lifetime analysis. PLoS One.

[R138] Watts D, Pulice RF, Reilly J, Brunoni AR, Kapczinski F, Passos IC (2022). Predicting treatment response using EEG in major depressive disorder: A machine-learning meta-analysis. Translational Psychiatry.

[R139] Woodham RD, Selvaraj S, Lajmi N, Hobday H, Sheehan G, Ghazi-Noori A-R, Lagerberg PJ, Rizvi M, Kwon SS, Orhii P, Maislin D (2025). Home-based transcranial direct current stimulation treatment for major depressive disorder: A fully remote phase 2 randomized sham-controlled trial. Nature Medicine.

[R140] World Health Organization (2017). Depression and other common mental disorders: Global health estimates. World Health Organization.

[R141] Xie L, Hu P, Guo Z, Chen M, Wang X, Du X, Li Y, Chen B, Zhang J, Zhao W, Liu S (2024). Immediate and long-term efficacy of transcranial direct current stimulation (tCDS) in obsessive-compulsive disorder, post-traumatic stress disorder and anxiety disorders: A systematic review and meta-analysis. Translational Psychiatry.

[R142] Xie Y, Guan M, Wang Z, Ma Z, Wang H, Fang P (2023). Alterations in brain connectivity patterns in schizophrenia patients with auditory verbal hallucinations during low frequency repetitive transcranial magnetic stimulation. Psychiatry Research.

[R143] Xiong Z, Tian C, Zeng X, Huang J, Wang R (2021). The relationship of functional connectivity of the sensorimotor and visual cortical networks between resting and task states. Frontiers in Neuroscience.

[R144] Yang F, Fang X, Tang W, Hui L, Chen Y, Zhang C, Tian X (2019). Effects and potential mechanisms of transcranial direct current stimulation (tDCS) on auditory hallucinations: A meta-analysis. Psychiatry Research.

[R145] Yeo BTT, Krienen FM, Sepulcre J, Sabuncu MR, Lashkari D, Hollinshead M, Roffman JL, Smoller JW, Zöllei L, Polimeni JR, Fischl B (2011). The organization of the human cerebral cortex estimated by intrinsic functional connectivity. Buckner.

[R146] Young IM, Dadario NB, Tanglay O, Chen E, Cook B, Taylor HM, Crawford L, Yeung JT, Nicholas PJ, Doyen S, Sughrue ME (2023). Connectivity model of the anatomic substrates and network abnormalities in major depressive disorder: A coordinate meta-analysis of resting-state functional connectivity. Journal of Affective Disorders Reports.

[R147] Yu J, Xie M, Song S, Zhou P, Yuan F, Ouyang M, Wang C, Liu N, Zhang N (2022). Functional connectivity within the frontal–striatal network differentiates checkers from washers of obsessive-compulsive disorder. Brain Sciences.

[R148] Yu L, Fang X, Chen Y, Wang Y, Wang D, Zhang C (2020). Efficacy of transcranial direct current stimulation in ameliorating negative symptoms and cognitive impairments in schizophrenia: A systematic review and meta-analysis. Schizophrenia Research.

[R149] Zeng L-L, Shen H, Liu L, Wang L, Li B, Fang P, Zhou Z, Li Y, Hu D (2012). Identifying major depression using whole-brain functional connectivity: A multivariate pattern analysis. Brain.

